# The Influence of Hydrophobic Blocks of PEO-Containing Copolymers on Glyceryl Monooleate Lyotropic Liquid Crystalline Nanoparticles for Drug Delivery

**DOI:** 10.3390/polym13162607

**Published:** 2021-08-05

**Authors:** Aleksander Forys, Maria Chountoulesi, Barbara Mendrek, Tomasz Konieczny, Theodore Sentoukas, Marcin Godzierz, Aleksandra Kordyka, Costas Demetzos, Stergios Pispas, Barbara Trzebicka

**Affiliations:** 1 Centre of Polymer and Carbon Materials, Polish Academy of Sciences, 34 ul. M. Curie-Skłodowskiej, 41-819 Zabrze, Poland; aforys@cmpw-pan.edu.pl (A.F.); bmendrek@cmpw-pan.edu.pl (B.M.); tkonieczny@cmpw-pan.edu.pl (T.K.); tsentoukas@cmpw-pan.edu.pl (T.S.); mgodzierz@cmpw-pan.edu.pl (M.G.); akordyka@cmpw-pan.edu.pl (A.K.); 2 Section of Pharmaceutical Technology, Department of Pharmacy, School of Health Sciences, National and Kapodistrian University of Athens, Panepistimioupolis Zografou, 15771 Athens, Greece; mchountoules@pharm.uoa.gr (M.C.); demetzos@pharm.uoa.gr (C.D.); 3 Theoretical and Physical Chemistry Institute, National Hellenic Research Foundation, 48 Vassileos Constantinou Avenue, 11635 Athens, Greece; pispas@eie.gr

**Keywords:** amphiphilic block copolymers, lipidic lyotropic liquid crystals, cryogenic transmission electron microscopy, fast Fourier transform, dynamic light scattering, X-ray diffraction, resveratrol, lipid/polymer carriers

## Abstract

The investigation of properties of amphiphilic block copolymers as stabilizers for non-lamellar lyotropic liquid crystalline nanoparticles represents a fundamental issue for the formation, stability and upgraded functionality of these nanosystems. The aim of this work is to use amphiphilic block copolymers, not studied before, as stabilizers of glyceryl monooleate 1-(cis-9-octadecenoyl)-*rac*-glycerol (GMO) colloidal dispersions. Nanosystems were prepared with the use of poly(ethylene oxide)-*b*-poly(lactic acid) (PEO-*b*-PLA) and poly(ethylene oxide)-*b*-poly(5-methyl-5-ethyloxycarbonyl-1,3-dioxan-2-one) (PEO-*b*-PMEC) block copolymers. Different GMO:polymer molar ratios lead to formulation of nanoparticles with different size and internal organization, depending on the type of hydrophobic block. Resveratrol was loaded into the nanosystems as a model hydrophobic drug. The physicochemical and morphological characteristics of the prepared nanosystems were investigated by dynamic light scattering (DLS), cryogenic transmission electron microscopy (cryo-TEM), fast Fourier transform (FFT) analysis and X-ray diffraction (XRD). The studies allowed the description of the lyotropic liquid crystalline nanoparticles and evaluation of impact of copolymer composition on these nanosystems. The structures formed in GMO:block copolymer colloidal dispersions were compared with those discussed previously. The investigations broaden the toolbox of polymeric stabilizers for the development of this type of hybrid polymer/lipid nanostructures.

## 1. Introduction

Lipids, as a primary component of liposomal drug delivery systems, have been at the focus of scientific interest owing to their unique self-assembly features. Among the numerous nanostructures formed by lipids, lyotropic liquid crystals (LLC) or liquid crystalline nanoparticles (LCNPs) with highly ordered internal structure are especially important objects for research [1,2,3]. According to the type of internal organization, LLC can generally be classified into three categories: lamellar phase (L_α_), cubic phase (V_2_) and hexagonal phase (H_2_), while the dispersion of each phase is known as liposomes, cubosomes and hexosomes, respectively [4,5,6]. Cubic and hexagonal phase have received wide interest due to their physicochemical properties and capability of encapsulating different types of compounds—from hydrophilic, through hydrophobic to amphiphilic. Combined with other properties like biocompatibility, nontoxicity and biodegradability, LLC have become an ideal potential platform for the design of versatile peptide [7] and drug delivery systems [8,9,10,11,12,13,14,15] as well as vaccines [16], imaging [17,18] and theranostics [19,20,21]. 

Various types of amphiphilic lipids are reported for the formation of lyotropic liquid crystals, such as phytantriol (PHYT) [16,22], glyceryl monooleate (GMO) [23,24], gadolinium oleate/myverol [17] and algal biomass [25]. To form stable LCNPs, aqueous dispersions of lipids require the addition of stabilizers, such as surfactants or amphiphilic block copolymers, which are one of the main class of polymeric stabilizers for these nanosystems [9,26]. It has been proven that the copolymers play a key role on the resulting formulation and determining the mesophases of final structures, while it also prevents unfavorable interactions between nanoparticles, such as aggregation [27,28]. In this case, hydrophilic block should be on the outer rim in the role of a hydrophilic corona, while the hydrophobic blocks should be anchored inside the lipid bilayer, forming hydrophobic interactions with the hydrophobic lipid tails. A schematic representation of the hybrid lipid/copolymer nanostructure can be found in [29,30]. Up until now, poly(ethylene oxide)-*b*-poly(propylene oxide)-*b*-poly(ethylene oxide) (PEO-PPO-PEO) triblock copolymers have been extensively used as stabilizers in these LLC systems, due to their biocompatibility and “stealth” properties [19,20,31]. However, the polymeric stabilizers can be carefully designed to provide extra properties that are extremely important due to the potential applications of lipid-based LLC for the drug delivery systems, such as stimuli-responsiveness [26,32,33]. In recent years, the finding of alternative amphiphilic block copolymers has been developing very fast, but the selection process of stabilizers is difficult, since it depends on many factors such as chemical structure, molar mass and hydrophobic–hydrophilic ratio of the chosen polymers [26,33,34]. 

One of the most popular lipids is glyceryl monooleate (GMO) [2,8,26,35] which is a polar, unsaturated monoglyceride. It can self-assemble into different mesophases, depending on water concentration and temperature [8,27,28]. Glyceryl monooleate is a nontoxic, biodegradable and biocompatible material, through it is commonly used for the preparation of liquid crystalline nanoparticles for drug delivery systems [8,9,10]. The amphiphilic stabilizer is necessary, in order for GMO dispersions to be created, because it provides steric stabilization by having its hydrophobic portion anchored in the lipid bilayers, while its more hydrophilic ends extend into the surrounding solution. In doing so, the inner cubic or hexagonal phase structure is maintained [36,37].

The most often used stabilizers in LLC systems are based on poly(ethylene oxide) (PEO), which is a polyether composed of repeated ethylene glycol units [-(CH_2_CH_2_O)_n_]. PEO is hydrophilic, non-ionic, non-toxic, non-immunogenic and biocompatible polymer. It is well investigated and has many potential applications, from industrial manufacturing, through chemistry to medicine and drug delivery [38,39,40,41]. The widespread use of PEO owes to the broad range of its possible molar mass and solubility in aqueous media, as well as in many organic solvents. Furthermore, high polarity of PEO, increases hydrophilicity and thus enhances water solubility of PEO containing structures [41,42,43,44]. It can be combined with many hydrophobic chains towards the development of block copolymers.

Another promising polymer reported for biomedical applications is poly(lactic acid) (PLA) which has been used extensively since the 1970s. It is comprised of biodegradable, aliphatic polyesters, derived from lactide or 2-hydroxy propionic acid, which is generally obtained by bacterial fermentation of carbohydrates from renewable sources, like agricultural crops such as corn, potato and cassava [45]. PLA is biocompatible and safe [46,47] thermoplastic, high-strength, high-modulus polymer and is widely used in both industry and biomedicine [48,49]. Poly(5-methyl-5-ethyloxycarbonyl-1,3-dioxan-2-one) (PMEC) is biocompatibile, crystalline, degradable and resorbable material being a methylcarboxytrimethylene carbonate derivative. Due to its simple structure, it can be easily prepared from 2,2-bis (methylol)propionic acid (bis-MPA) in high yield and homopolymerized with predictable molar mass and narrow dispersity [50,51,52].

In the present study, novel potential polymeric stabilizers of glyceryl monooleate 1-(cis-9-octadecenoyl)-*rac*-glycerol lyotropic liquid crystals were investigated. More specifically, amphiphilic block copolymers were synthesized consisting of hydrophilic poly(ethylene oxide) and different hydrophobic blocks of poly(lactic acid) and poly(5-methyl-5-ethyloxycarbonyl-1,3-dioxan-2-one) (PMEC). We also compared its stabilizing ability with previously studied poly(ethylene oxide)-*b*-poly(ε-caprolactone) (PEO-*b*-PCL) block copolymers [28].

>The prepared nanosystems were analyzed using a gamut of techniques, such as dynamic light scattering (DLS) for the physicochemical characterization, cryogenic transmission electron microscopy (cryo-TEM), fast Fourier transform (FFT) analysis and X-ray diffraction (XRD) for the morphological evaluation.

Studied LCNPs served as nanocarriers of resveratrol, a model hydrophobic drug that is a well-studied biologically active compound and exhibits various pharmacological properties. Among the many, it can be distinguished as anti-oxidative, anti-inflammatory, neuro-protective, anti-aging and anticancer drug [28,53,54].

The drug was encapsulated in the liquid crystalline nanoparticles, and its influence on the resulting structures was also examined. The resveratrol carriers obtained in this study were compared with these previously prepared with the use of PEO-*b*-PCL as a GMO dispersion stabilizer.

To the best of our knowledge, this is the first report where the above referred block copolymers were used as stabilizers for liquid crystalline nanoparticles.

## 2. Materials and Methods

### 2.1. Materials

All liquid crystalline nanosystems were prepared from glyceryl monooleate lipid Monomuls^®^ 90-O18 (1-(cis-9-octadecenoyl)-*rac*-glycerol), (GMO) (BASF, Ludwigshafen, Germany) and used without further purification. All formulations were prepared in HPLC-grade water.

Resveratrol was acquired from Sigma-Aldrich Chemical Co (St. Louis, MO, USA). 

Dichloromethane (POCH) was dried over CaH_2_ and distilled under reduced pressure prior to use. THF (POCH) was distilled over a sodium-potassium alloy. Dowex 50WX8 (Sigma-Aldrich, Darmstadt, Germany) was washed with dry THF before use. Methoxy ether poly(ethylene oxide) 5000 (mPEO_123_-OH, TCI, M_n_(GPC) = 5 200, M_n_(NMR) = 5 400) was dried by two azeotropic distillations using anhydrous toluene. L-lactide (LA) (>99.5%, Forusorb) was purified by sublimation two times before use. 1,8-Diazabicyclo[5.4.0]undec-7-ene (DBU) (98%, Sigma-Aldrich) was distilled under reduced pressure over BaO. Triethylamine (TEA, >99%, Sigma-Aldrich) was distilled under reduced pressure over BaO. 2,2-Bis(hydroxymethyl)propionic acid (bis-MPA, >97%, TCI), ethanol (96%, POCH), ethyl chloroformate (97%, Sigma-Aldrich), Amberlyst 15 (Sigma-Aldrich) and magnesium sulphate (Chempur, Piekary Slaskie, Poland) were used as received.

### 2.2. Methods

#### 2.2.1. MEC Synthesis

Bis-MPA (44.2 g, 0.330 mol, 1.0 eq.) was dissolved in 300 mL of ethanol with 13.6 g of Amberlyst 15. Solution was refluxed for 24 h. Then, the resin was filtered, and unreacted ethanol was stripped off. The residue was dissolved in 400 mL of DCM and the insoluble part was removed via filtration. After removal of DCM, ethyl 2,2-bis(hydroxymethyl)propionate (bis-MPA-Et) was received as a colorless, viscous liquid. Yield: 75%.

In the second step, into the solution of bis-MPA-Et (40.0 g, 0.247 mol, 1 eq.) and ethyl chloroformate (58.7 mL, 0.617 mol, 2.5 eq.) in 400 cm^3^ of DCM at 0 °C, TEA (85.9 mL, 0.617 mol, 2.5 eq.) diluted with 100 mL of DCM was added dropwise over a period of 1 h. The reaction mixture was kept in dry nitrogen atmosphere at 0 °C for 2 h and then at room temperature for 24 h. Then, the mixture was filtered, and the filtrate was concentrated under vacuum. Crude product was dissolved in 100 mL of DCM and washed with 50 mL 1 M HCl_(aq)_ twice and 50 mL saturated solution of NaHCO_3_, 25 mL of brine and 25 mL of distilled water. The organic phase was dried with anhydrous MgSO_4_. Then, MgSO_4_ was filtered off and DCM was stripped off. Crude product was then purified by crystallization from ethyl acetate twice to give MEC, as white crystals. Yield: 39%.

#### 2.2.2. Polymerization of the Block Copolymers

**Figure 1:**
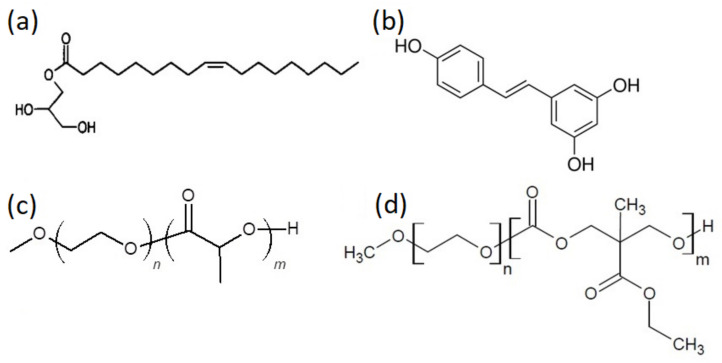
Chemical structures of (**a**) glyceryl monooleate (GMO) lipid, (**b**) resveratrol, (**c**) poly(ethylene oxide)-*b*-poly(lactic acid) (PEO-*b*-PLA), (**d**) poly(ethylene oxide)-*b*-poly(5-methyl-5-ethyloxycarbonyl-1,3-dioxan-2-one) (PEO-*b*-PMEC), employed in this study.

The chemical structures and the molecular characteristics of copolymer samples are presented in Figure 1 and in Table 1, respectively.

Polymerization of poly(ethylene oxide)-*b*-poly(lactic acid) (PEO-*b*-PLA) was performed in an anhydrous atmosphere (glove-box, H_2_O < 1 ppm, O_2_ < 3 ppm). The diblock copolymer was synthesized by ring-opening polymerization (ROP) of LA using mPEO_123_-OH as an initiator and DBU as catalyst in dry CH_2_Cl_2_ at the monomer concentration equal to 1.000 mmol/mL at room temperature.

Detailed polymerization procedures and chemical characterization of the block copolymers are presented in Supporting Information.

In case of poly(ethylene oxide)-*b*-poly(5-methyl-5-ethyloxycarbonyl-1,3-dioxan-2-one) (PEO-*b*-PMEC), polymerization was performed in an anhydrous atmosphere (glove-box, H_2_O < 1 ppm, O_2_ < 3 ppm). The diblock copolymer was synthesized by ring-opening polymerization (ROP) of MEC using mPEO_123_-OH as an initiator and DBU as catalyst in dry CH_2_Cl_2_ at monomer concentration equal to 1.000 mmol/mL at room temperature.

Detailed polymerization procedures and chemical characterization (Figures S1 and S2) of the block copolymers are summarized in Supporting Information.

#### 2.2.3. Preparation of Liquid Crystalline Nanoparticle Dispersions

PEO-*b*-PLA and PEO-*b*-PMEC copolymers were tested as polymeric stabilizers, each one individually. Two different weight ratios were prepared, namely GMO:polymeric stabilizer 9:1 and 4:1 respectively, which correspond to two different concentrations of the stabilizer, namely 10% and 20% *w*/*w* relative to the lipid mass. Copolymers were able to stabilize the lipid in nanoparticles in both lipid:polymer ratios. The lipid concentration was 20 mg/mL in all prepared systems. The temperature used during the preparation process of all the systems was 45 °C.

All liquid crystalline systems were prepared by Top-Down Method (TD). More specifically, GMO was weighted into glass vials and heated to 45 °C, until free flowing. The appropriate volume of HPLC-grade water solution (pH = 6.0), containing the different amounts of the polymeric stabilizers, was added to the vials containing the lipids, in order to achieve a lipid concentration of 20 mg/mL. The mixtures were firstly sonicated using a bath sonicator for 2 min, at 45 °C, followed by two 2-min sonication cycles (amplitude 70, cycle 0.7), interrupted by a 2-min resting period, using a probe sonicator (UW 2070 Bandelin electronic, Berlin, Germany), until a milky dispersion was formed. The resultant dispersions were allowed to anneal for 30 min, then stored at room temperature and measured 5 days after preparation.

In the case of liquid crystalline systems with entrapped resveratrol, the same process as above was followed, by the difference that the appropriate amounts of resveratrol and GMO were fully dissolved in ethanol initially. Ethanol was gently evaporated until a dry film of liquid-resveratrol mixture was achieved. The total resveratrol concentration in the final dispersion was 2 mg/mL.

#### 2.2.4. Physicochemical and Morphological Characterization of the Liquid Crystalline Dispersions

##### Dynamic and Electrophoretic Light Scattering

Hydrodynamic radius and zeta potential measurements were performed at least in triplicate on a Zetasizer Nano ZS 90 (Malvern Instruments, Malvern, Worcestershire, UK) in disposable cuvettes and processed with Zetasizer Software (Malvern Instruments, Malvern, Worcestershire, UK) version 6.32.

##### Cryogenic Transmission Electron Microscopy (Cryo-TEM)

Cryogenic Transmission Electron Microscopy (cryo-TEM) images were obtained using a Tecnai F20 X TWIN microscope (FEI Company, Hillsboro, OR, USA) equipped with field emission gun, operating at an acceleration voltage of 200 kV. Images were recorded on the Gatan Rio 16 CMOS 4k camera (Gatan Inc., Pleasanton, CA, USA) and processed with Gatan Microscopy Suite (GMS) software (Gatan Inc., Pleasanton, CA, USA). Specimen preparation was done by vitrification of the aqueous solutions on grids with holey carbon film (Quantifoil R 2/2; Quantifoil Micro Tools GmbH, Großlöbichau, Germany). Prior to use, the grids were activated for 15 s in oxygen plasma using a Femto plasma cleaner (Diener Electronic, Ebhausen, Germany). Cryo-samples were prepared by applying a droplet (3 μL) of the suspension to the grid, blotting with filter paper and immediate freezing in liquid ethane using a fully automated blotting device Vitrobot Mark IV (Thermo Fisher Scientific, Waltham, MA, USA). After preparation, the vitrified specimens were kept under liquid nitrogen until they were inserted into a cryo-TEM-holder Gatan 626 (Gatan Inc., Pleasanton, CA, USA) and analyzed in the TEM at −178 °C.

##### X-ray Diffraction (XRD)

Samples for X-ray diffraction studies were prepared by evaporation of H_2_O at 50 °C, up to a final concentration around 8–10 mg/mL. Samples obtained from dispersions were placed between Capton foil in PMMA holder with additional PMMA 1 mm thick distance, in order to obtain optimal intensity of scattering in water.

XRD was performed using the D8 Advance diffractometer (Bruker, Karlsruhe, Germany) with Cu-Kα cathode (λ = 1.54 nm) working in transmission mode. The scan rate was 0.02°/min with scanning step 0.01° in range of 0.5° to 3° 2Θ. All patterns were acquired at least seven times, then accumulated to obtained higher resolution. Background subtraction was performed using DIFFRAC.EVA program, using air scattering filter. Obtained patterns were smoothed using Fourier smooth filter. Typically, *Im3m* and *Pn3m* cubic structures are reported for this type of liquid crystalline, according to literature [36,55,56,57]. Model patterns of two cubic, hexoctahedral phases: body centered *Im3m* and primitive *Pn3m* were calculated using DIFFRAC.EVA program, assuming that lattice parameter a = 130 Å [55,57], while exact lattice parameters of fitted phase were calculated using Rietveld refinement in TOPAS 6 program, based on Williamson-Hall theory. The pseudo-Voigt function was used in the description of diffraction line profiles at the Rietveld refinement [58,59]. The R_wp_ (weighted-pattern factor) and GOF (goodness-of-fit) parameters were used as numerical criteria of the quality of the fit of calculated to experimental diffraction data.

#### 2.2.5. Characterization of the Liquid Crystalline Dispersions with Entrapped Resveratrol

##### Entrapment Efficiency and Drug Loading Determination

The ultrafiltration centrifugal method was utilized for the separation of the free resveratrol from the resveratrol entrapped in the liquid crystalline nanoparticles [15,23]. The liquid crystalline nanoparticle dispersions were centrifuged for 45 min at 8000 rpm inside the centrifugal filter tubes, with a molecular weight cutoff of 10 kDa, at 4 °C. The nanoparticles were separated from the aqueous phase and the supernatant was analyzed via UV-Vis spectroscopy in order to quantitate the free resveratrol concentration. Absorption measurements were performed at a range of 190–600 nm, while the value at 307 nm and a pre-constructed calibration curve were used for the analysis. In addition, plain liquid crystalline nanoparticles were centrifuged, and supernatant was used as blank. The entrapment efficiency (EE)% was calculated using the following equation:

$$\left(EE\right)\;\%=(1-\frac{C_{supernatant}}{C_{total}})\;\%$$

where *C_supernatant_* is the resveratrol concentration that was quantified in the supernatant (non-entrapped) and *C_total_* is the total concentration of the resveratrol added in the initial hybrid liposomes aqueous solutions (2 mg/mL) [15,23,31].

The drug loading (DL)% was calculated according to the following equation

$$\left(DL\right)\;\%=(\frac{C_{total}-C_{supernatant}}{C_{lipid}})\;\%$$

where *C_total_* is the total concentration of the resveratrol added in the dispersion (2 mg/mL), *C_supernatant_* is the non-entrapped resveratrol concentration and *C_lipid_* is the total concentration of the lipid in the aqueous solution (20 mg/mL) [15,31].

##### In Vitro Resveratrol Release Studies

The drug-release studies were conducted using the dialysis method in the dark, [15,23,31,60,61] based on a previous study [28]. 0.5 mL of the aqueous solution samples were placed into a dialysis sack, with a molecular weight cutoff at 10,000. The dialysis sacks were inserted in a 20 mL PBS (pH = 7.4) shaking water bath set at 37 °C. Aliquots of samples were taken from the external solution at specific time intervals, while the volume was replaced with fresh release medium. Samples were diluted with ethanol and quantified for drug content by an Analytik Jena Specord 200 plus UV-Vis spectrometer (Jena, Thuringia, Germany), in the range of 190–600 nm. The values corresponding at 307 nm of the spectra were used for the analysis, along with a pre-constructed standard curve for resveratrol in PBS/ethanol. In addition, empty hybrid liposomes were used as blanks. The % cumulative mass of resveratrol released versus time was plotted. 

## 3. Results and Discussion

### 3.1. Physicochemical and Morphological Characteristics of the Liquid Crystalline Nanosystems as Revealed by DLS and Cryo-TEM

The chemical composition of amphiphilic polymeric stabilizers PEO-*b*-PLA and PEO-*b*-PMEC affected the size, as well as the morphology of prepared nanosystems. Both block copolymers used in our study were found to provide stable GMO-based colloidal dispersions, at least at the studied ratios. Samples were measured 5 days after preparation. We assume that the present nanosystems were stable as it was in the case of previously studied GMO:PEO-*b*-PCL [28] and as shown for Pluronic F127 systems [27,62].

The GMO:copolymers hydrodynamic radius and size dispersity (PDI) measured by DLS and average sizes from cryo-TEM images are given in Table 2. Cryo-TEM histograms are presented in Supporting Information (Figures S3 and S4). Size distribution by intensity from DLS are shown in Supporting Information (Figures S5 and S6).

Starting from the GMO:PEO-*b*-PLA systems, an increase of the total amount of copolymer (from 9:1 to 4:1 w/w) led to an increase of the particle size. Nanosystems with more stabilizer content are more homogenous, exhibiting smaller values of PDI, with negative ζ-potential values for both ratios.

**Figure 2:**
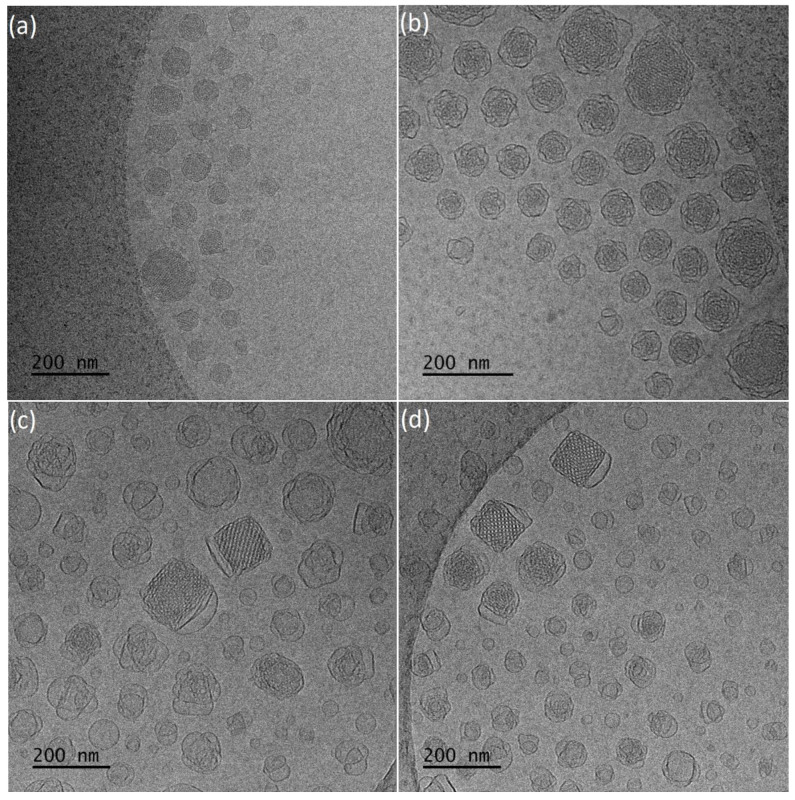
Representative cryo-TEM images of the (**a**) GMO:PEO-*b*-PLA 9:1, (**b**) GMO:PEO-*b*-PLA 4:1, (**c**) GMO:PEO-*b*-PMEC 9:1, (**d**) GMO:PEO-*b*-PMEC 4:1.

**Figure 3:**
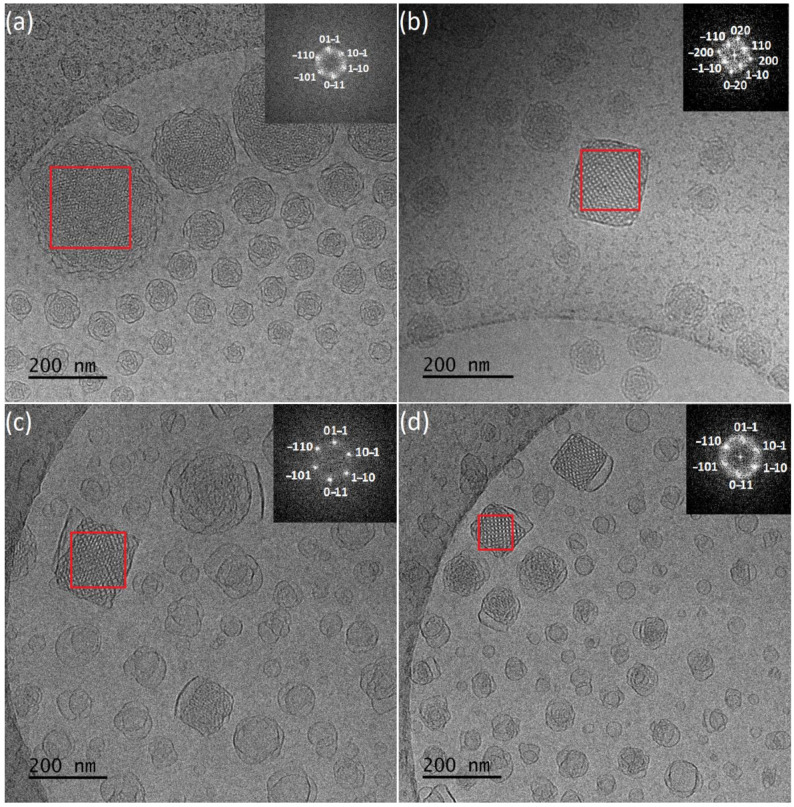
Fast Fourier transform analysis of particles with ordered internal structure for (**a**) GMO:PEO-*b*-PLA 9:1, (**b**) GMO:PEO-*b*-PLA 4:1, (**c**) GMO:PEO-*b*-PMEC 9:1, and (**d**) GMO:PEO-*b*-PMEC 4:1.

Cryo-TEM revealed morphological variety inside the dispersions (Figure 2a,b). More specifically, in the first case with a smaller amount of PEO-*b*-PLA, multilamellar particles prevailed, with confined, striated, curved inner structure, resembling an onion. This large population was coexisting with a small number of “sponge-like” nanoparticles, consisting of an outer layer built of intersecting lamellas and an apparent dense inner core with highly disordered interior and with absence of long-range order and periodicity [63,64], and particles with a highly ordered internal structure. FFT analysis of images (Figure 3a) gave a space group close to *Pn3m* (double-diamond type bicontinuous cubic phase). Simple vesicles were not observed by cryo-TEM.

In the case of GMO:PEO-*b*-PLA 4:1 ratio, cryo-TEM revealed the predominant spherical, “sponge-like” particles, and particles with a highly ordered internal structure. The fast Fourier transform analysis (Figure 3b) gave a space group symmetry likely to *Im3m* (primitive type bicontinuous cubic phase). In addition, particles with *Pn3m* symmetry were observed (Figure S7). Apart from this category of LCNPs with ordered internal structure, there were also spherical vesicles observed as a minority component.

A small number of large aggregates with irregular shape were observed for both GMO:PEO-*b*-PLA ratios.

**Figure 4:**
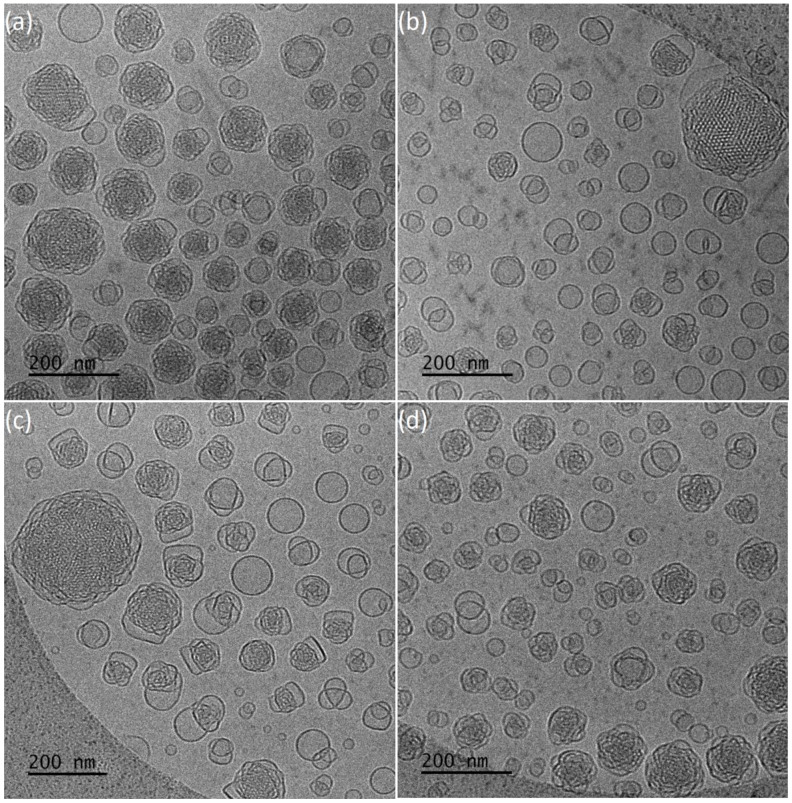
Representative cryo-TEM images of the (**a**) GMO:PEO-*b*-PLA 9:1 + resveratrol, (**b**) GMO:PEO-*b*-PLA 4:1 + resveratrol, (**c**) GMO:PEO-*b*-PMEC 9:1 + resveratrol, (**d**) GMO:PEO-*b*-PMEC 4:1 + resveratrol.

The encapsulation of hydrophobic resveratrol affected the physicochemical and morphological characteristics of the prepared nanosystems. The average size of particles is higher in the case of 9:1 ratio and smaller for 4:1, compared to those without the drug (Fig.S3 and S4). They present negative ζ-potential values and smaller size dispersity (Table 2). According to cryo-TEM results, systems with resveratrol (Figure 4a,b) are characterized by significant differences in particles morphology. For 4:1 ratio, a population of spherical vesicles and “sponge-like” particles was observed. Moreover, elongated structures that were not found in drug-free systems were observed. Smaller amount of copolymer results predominant in “sponge-like” particles, coexisting with small number of spherical vesicles. Worm-like structures were in minority.

Both GMO:PEO-*b*-PLA ratios systems containing resveratrol resulted in particles of unorganized internal structure, which was assessed with FFT analysis.

Replacement PEO-*b*-PLA with PEO-*b*-PMEC with bigger side group in the hydrophobic blocks, lead to a different, prevailing morphology of nanosystems (Figure 2c,d). Average particles size from DLS and cryo-TEM is higher in case of 9:1, compared to GMO:PEO-*b*-PLA. For 4:1 ratio, average DLS results are similar, but cryo-TEM histograms reveal smaller sizes of particles. A higher amount of this stabilizer caused a decrease of the particle size, compared to a 9:1 ratio, probably because of the larger reduction of interfacial tension between GMO lipid and water phase. Consequently, it led to the formation of a larger surface area and fragmentation of particles towards smaller sizes [32,36,65,66].

Nanosystems were found to present PDI values of 0.55 and 0.63, respectively, with negative ζ-potential values for both ratios (Table 2).

Vesicles, “sponge-like” particles and particles with organized internal structure and cubic shape were observed at both ratios. The fast Fourier transform analysis (Figure 3c,d) gave a space group symmetry likely to *Pn3m*. The increase of the PEO-*b*-PMEC concentration caused decrease of the nanoparticles with regular organization and led to the formation of higher amount of small, simple vesicles exhibiting no internal structure and irregularly shaped particles, which may represent different stages of the fusion processes that are taking place towards the formation of the more organized structures.

This is in accordance with the literature [32,56] which confirms that higher stabilizer concentrations can cause formation of vesicular structures, such as liposomes, reducing the percentage of the existing cubic structures.

The addition of resveratrol results in higher average sizes of particles, compared to pure nanosystems. PDI values are higher for the 4:1 ratio and smaller for the second ratio (Table 2). Cryo-TEM (Figure 4c,d) revealed differences in morphology of the particles for these ratios. Predominantly “sponge-like” structures were observed for higher amount of copolymer with drug present. This large population was coexisting with spherical vesicles. In the case of the 9:1 ratio, the vesicles population was increased compared to “sponge-like” structures.

**Figure 5:**
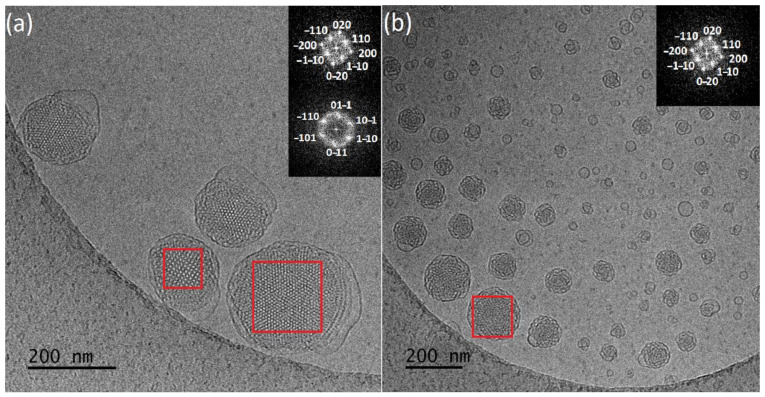
Fast Fourier transform (FFT) analysis of particles with ordered internal structure for (**a**) GMO:PEO-*b*-PMEC 9:1 + resveratrol, and (**b**) GMO:PEO-*b*-PMEC 4:1 + resveratrol.

Apart from these categories of structures, there were also particles with ordered internal structure, observed for both ratios in minority. FFT analysis gave a space group close to *Im3m* (primitive type bicontinuous cubic phase) (Figure 5a,b) and *Pn3m* (double-diamond type bicontinuous cubic phase) (Figure 5a).

We have compared the nanosystems of GMO prepared with PEO-*b*-PCL from our previous work [28] with the same PEO block and similar molar mass as the copolymers used here. All PEO-*b*-PCL systems were found to present PDI values ≤ 0.25, with negative ζ-potential values. Higher amount of PEO-*b*-PCL copolymer (4:1 ratio) yielded smaller nanoparticles, similar to GMO:PEO-*b*-PMEC nanosystems. It can be observed that the chemical composition of the used copolymer influenced the size of the nanoparticles. PEO-*b*-PCL caused formation of particles with smaller average dimension than PEO-*b*-PLA and PEO-*b*-PMEC. Furthermore, nanosystems are more homogenous exhibiting smaller values of PDI compared to new ones. All GMO:copolymers systems present negative ζ-potential values.

In addition, significant differences in particles morphology were revealed by cryo-TEM. More specifically, spherical vesicles were coexisting with liquid crystalline nanoparticles with lower grades of internal organization that resemble “sponge-like” structures, for both ratios. Higher concentration of PEO-*b*-PCL results in absence of nanoparticles with ordered internal structure, as confirmed with fast Fourier transform analysis. GMO:PEO-*b*-PCL 9:1 systems exhibited nanoparticles with a highly ordered internal structure, being square or almost squared shaped. FFT analysis patterns give body centered *Im3m* symmetry.

### 3.2. X-ray Diffraction

**Figure 6:**
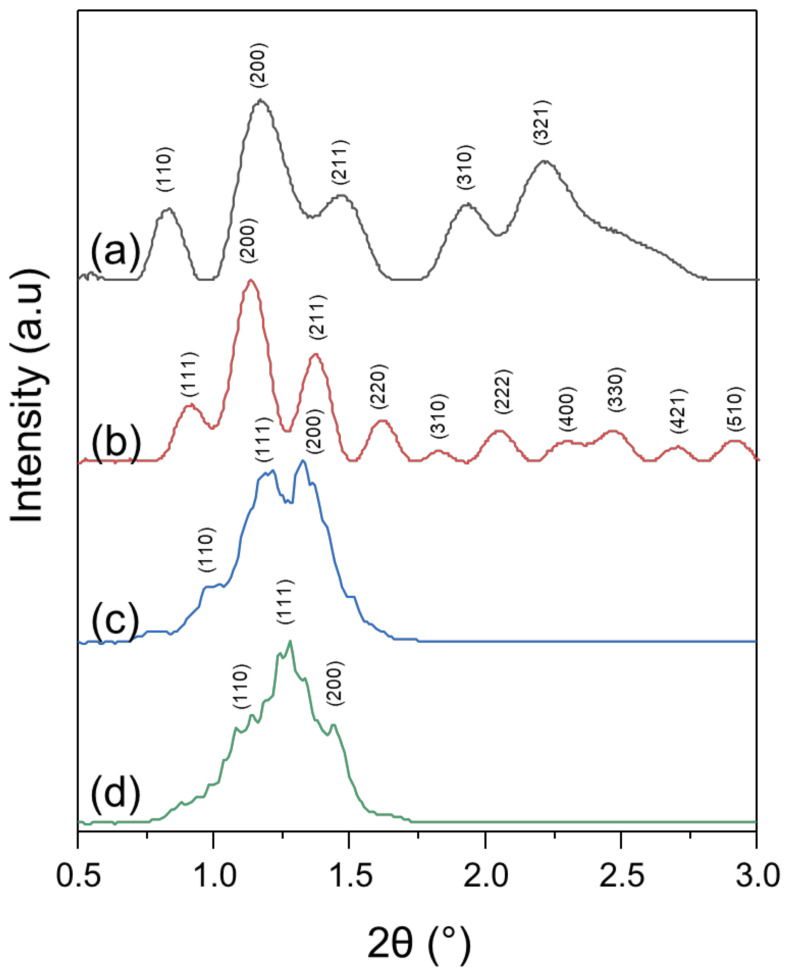
XRD patterns with marked peaks of fitted space groups of (**a**) GMO:PEO-*b*-PLA 4:1, (**b**) GMO:PEO-*b*-PLA 9:1, (**c**) GMO:PEO-*b*-PMEC 4:1, (**d**) GMO:PEO-*b*-PMEC 9:1.

The organized internal structure of particles, assessed with fast Fourier transform analysis is likely corresponding to cubic structures of *Pn3m* or *Im3m* symmetry [67]. For the full characterization of the predominant ordered internal structure (Table 2), samples were measured by X-ray diffraction (Figure 6).

For GMO:PEO-*b*-PLA, the fitting of calculated theoretical structure patterns gives slightly better fit for *Im3m* structure than *Pn3m* in the case of 4:1 stoichiometry. However, considering peak positions and corresponding d-spacings, it should be noticed that for primitive *Pn3m* the (111) peak is missing, which results in worse fit for that structure. Further fitting with Rietveld refinement of the assumed body centered *Im3m* structure shows lattice parameter a = 148.4 ± 0.7 Å. In the case of 9:1 sample, better fit was obtained for primitive *Pn3m* cubic structure with lattice parameter a = 151.1 ± 0.9 Å. Calculated lattice parameters of GMO:PEO-*b*-PLA shows lattice enlargement in comparison to the liquid crystals described in the literature [36,55,56,57].

In case of GMO:PEO-*b*-PMEC nanosystems, the characteristic (111) peak of *Pn3m* structure was observed (Figure 6). Rietveld refinement confirms presence of *Pn3m* structure for 4:1 and 9:1 nanosystems with lattice parameters 129.6 ± 0.6 and 131.3 ± 0.7 Å, respectively. Moreover, peak shape and intensity are more developed in the case of the 9:1 nanosystem than for the 4:1 ratio.

Comparing all of the examined samples, the best well-developed structure was observed for the GMO:PEO-*b*-PLA 9:1 nanosystem, while low ordered structure was detected for the GMO:PEO-*b*-PMEC 4:1 nanosystem. In both copolymers some regularity was detected—higher amount of GMO lipid (9:1) allows higher order of the obtained structure. In the GMO:PEO-*b*-PLA 4:1 system—a body centered *Im3m* structure was observed instead of primitive *Pn3m*. *Pn3m* structure was reported in literature for PEO-PPO-PEO liquid nanoparticles [36,67] and in our studies for PEO-*b*-PCL cubosomes [28].

### 3.3. Resveratrol Entrapment and In Vitro Release Studies

In order to investigate the ability of the above lipid:copolymer hybrid nanosystems to encapsulate drug molecules and act as drug nanocarriers, the hydrophobic drug resveratrol was employed as a low molecular weight cargo molecule model.

**Figure 7:**
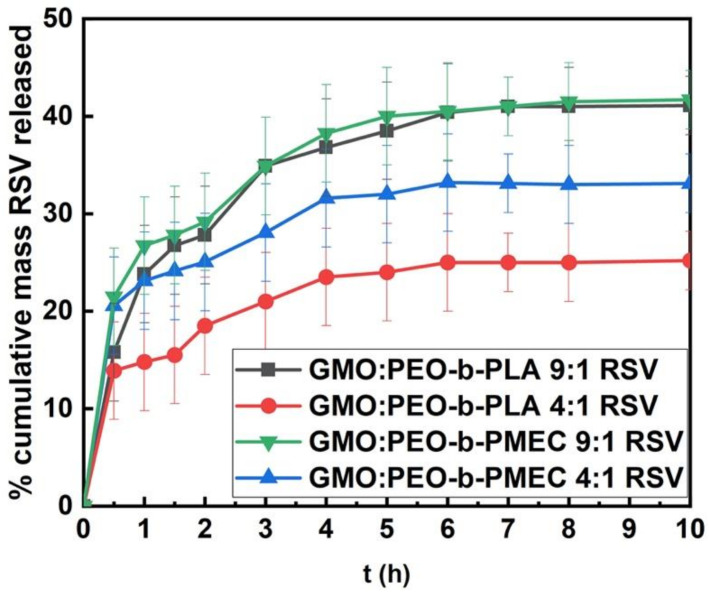
Cumulative mass of resveratrol that is released (%) from the hybrid liquid crystalline nanoparticles (±SD values are given for 5 runs).

Resveratrol was incorporated to GMO:PEO-*b*-PLA and GMO:PEO-*b*-PMEC LCNPs to test their drug-loading and release abilities in conditions similar to those inside of a human body. The results of loading and entrapment efficiency are shown in Table 3. The entrapment efficiency was over 99% for resveratrol for all systems under study. The release of the drug from its carriers takes place in three stages as shown in Figure 7. A quick resveratrol release in terms of 10–25% approximately of its total mass can be seen for the first half-hour, probably due to the large surface area to volume ratio of the nanoparticles [23]. A prolonged release is observed for the next three hours which is associated with the strong entrapment of the drug inside the nanosystem [68,69]. Finally, a plateau was observed with no extra drug-release, with the total released resveratrol mass under 40%. This is a typical behavior for such liquid crystalline nanosystems [70,71]. Resveratrol, as a hydrophobic drug can “hide” deeply inside the GMO lipid bilayer, as proved by the high entrapment efficiency percentages. The prolonged release is driven by certain factors, such as the “pore” size [3,72], the organization of the cubic phase network and the entrapped water phase. They are crucial for the diffusion of the drug to the aqueous medium through the “water channels”. Thus, highly organized networks lead in tighter nanostructures that result in more drug retention, while looser formations lead to maximum drug release [23,31].

The differences shown in the release profile graphs in Figure 7 can be attributed to the block copolymers, which strongly influence the formation of the final nanostructures. The pore size and tortuosity of the water channels of the cubic phase contribute to the sustained release because the entrapped molecule has to be navigated through a nano-tubular aqueous network assembled by the continuous lipid bilayer architecture.

All the under-study LCNPs are highly influenced by resveratrol since its encapsulation results in the formation of nanostructures with lower internal order. Vesicles and “sponge-like” nanoparticles prevail when the drug is loaded in comparison to the “empty” ones, as shown in cryo-TEM studies. It is possible that resveratrol, being the most hydrophobic molecule in these systems, antagonizes PMEC and PLA in terms of entrapment inside the GMO phase, altering the final nanostructures.

The GMO:PEO-*b*-PLA 4:1 nanosystem presented a minimum release of resveratrol, depicting a more complex network than the other systems, preventing the drug to “find-its-way” to the external aqueous medium. On the other hand, both GMO:PEO-*b*-PLA and GMO:PEO-*b*-PMEC 9:1 showed the maximum resveratrol release, with an almost identical behavior, probably due to a looser internal structure. Last but not least, drug release from the GMO:PEO-*b*-PMEC 4:1 nanoparticles was somewhere in the middle of all four systems. Such differences in the drug-release behavior of each system are driven by the ratio of the diblock copolymers and the variations in the nature of hydrophobic units. It should be emphasized that the copolymer molar masses, and hydrophobic units in the chains were nearly identical.

Previously studied GMO:poly(ethylene oxide)-*b*-poly(ε-caprolactone) and GMO:poly(2-methyl-2-oxazoline)-*grad*-poly(2-phenyl-2-oxazoline) (MPO_x_) LCNPs [28] showed a similar behavior in terms of initial, prolonged and plateau release profile stages. All the systems presented excellent entrapment efficiency, over 97%, which is attributed to the lipophilicity of resveratrol and GMO bilayers formed in chimeric particles. In the case of GMO:PEO-*b*-PCL and GMO:MPOx, the prolonged release lasted more than 3 h. Conditions used in the present study were the same as in a previous studied GMO:PEO-*b*-PCL+resveratrol system (Phosphate Buffer Saline, PBS) [28]. This allowed for the comparison between the systems.

The released amount of resveratrol for the GMO:PEO-*b*-PMEC and GMO:PEO-*b*-PLA LCNPs is smaller in terms of % cumulative mass in respect with the GMO:PEO-*b*-PCL and GMO:MPO_x_. The release from hybrid particles can be related to the internal network phase, its complexity and organization. In the studied systems the ceiling release is lower than for GMO:PEO-*b*-PCL which also indicates that in the present case structures are better organized for preventing drug release.

## 4. Conclusions

Non-lamellar lipid liquid crystalline nanosystems were prepared from GMO and the PEO-*b*-PLA and PEO-*b*-PMEC block copolymers at different GMO:copolymer ratios (9:1 and 4:1). The copolymers were obtained using the same PEO macroinitiator and had hydrophobic blocks of similar weight. All block copolymers were found to act successfully as stabilizers and provide GMO-based colloidal dispersions at the studied ratios. The influence of hydrophobic blocks of the PEO-containing copolymers was investigated in physicochemical and morphological terms. Different GMO and copolymer molar ratios lead to formulation of nanoparticles with different size and internal organization. The hydrophobic block of the copolymers affected the size and morphology of the nanosystems. The nanosystems stabilized by the PEO-*b*-PMEC copolymer exhibited higher grades of internal organization with respect to PEO-*b*-PLA, as revealed by cryo-TEM, fast Fourier transform and X-ray diffraction analysis. They appeared to be better organized than previously studied stabilizer PEO-*b*-PCL.

The loading of hydrophobic resveratrol influenced the morphology and internal organization of the final hybrid lipid-polymer nanostructures with incorporated drug.

These findings support the use of new, alternative polymeric stabilizers for preparation of LCNPs, also with potential applications as the drug delivery systems, broadening the already existing toolbox of polymeric stabilizers and hybrid lipid-copolymer structures.

## Figures and Tables

**Table 1:** Molecular characteristics of copolymers utilized.

Polymer	DP_(NMR)_ of Hydrophobic Block	M_n(NMR)_	M_w(GPC)_	M_w_/M_n_ ^a^	%wt ^b^
**PEO-*b*-PLA**	18	6700	6850	1.06	%wt PEO 81
**PEO-*b*-PMEC**	9	7100	6560	1.05	%wt PEO 76

^a^ By SEC in DMF, calculated using PEO standards calibration; ^b^ by ^1^H-NMR in CDCl_3_.

**Table 2:** Dynamic light scattering, ζ-potential, cryo-TEM histograms, XRD and FFT analysis results.

Sample	Weight Ratio	*R_h_* (nm)	PDI (±SD)	ζ-Pot (mV)	Average Size (nm) Cryo-TEM	Space Group XRD	Space Group FFT
**GMO:** **PEO-*b*-PLA**	9:1	88 and 10	0.53 (±0.04)	−13	66	*Pn3m*	*Pn3m*
**GMO:** **PEO-*b*-PLA**	4:1	137 and 18	0.62 (±0.01)	−17	88	*Im3m*	*Im3m/Pn3m*
**GMO:** **PEO-*b*-PMEC**	9:1	150 and 19	0.63 (±0.03)	−21	85	*Pn3m*	*Pn3m*
**GMO:** **PEO-*b*-PMEC**	4:1	141 and 16	0.55 (±0.02)	−16	63	*Pn3m*	*Pn3m*
**GMO:** **PEO-*b*-PLA** **+resveratrol**	9:1	17	0.40 (±0.02)	−26	85	-	-
**GMO:** **PEO-*b*-PLA** **+resveratrol**	4:1	146 and 20	0.55 (±0.04)	−13	78	-	-
**GMO:** **PEO-*b*-PMEC** **+resveratrol**	9:1	179 and 23	0.58 (±0.01)	−27	147	-	*Im3m/Pn3m*
**GMO:** **PEO-*b*-PMEC** **+resveratrol**	4:1	157 and 14	0.85 (±0.04)	−18	57	-	*Im3m*

**Table 3:** Drug loading and entrapment efficiency results.

Sample	Weight Ratio	Entrapment Efficiency (%)	Drug Loading (%)
**GMO:PEO-*b*-PLA**	9:1	99.91	9.99
**GMO:PEO-*b*-PLA**	4:1	99.84	9.98
**GMO:PEO-*b*-PMEC**	9:1	99.84	9.98
**GMO:PEO-*b*-PMEC**	4:1	99.46	9.94

## Data Availability

The data presented in this study are available on request from the corresponding author.

## Supplementary Materials

The following are available online at https://www.mdpi.com/article/10.3390/polym13162607/s1, Figure S1. ^1^H NMR spectra of the (a) PEO-*b*-PLA, (b) PEO-*b*-PMEC, Figure S2. GPC chromatogram of the (a) PEO-*b*-PLA, (b) PEO-*b*-PMEC, Figure S3. Cryo-TEM histograms of the (a) GMO:PEO-*b*-PLA 9:1, (b) GMO:PEO-*b*-PLA 4:1, (c) GMO:PEO-*b*-PMEC 9:1, (d) GMO:PEO-*b*-PMEC 4:1 nanosystem, Figure S4. Cryo-TEM histograms of the of the (a) GMO:PEO-*b*-PLA 9:1 + resveratrol, (b) GMO:PEO-*b*-PLA 4:1 + resveratrol, (c) GMO:PEO-*b*-MEC 9:1 + resveratrol, (d) GMO:PEO-*b*-MEC 4:1 + resveratrol nanosystems, Figure S5. Size distributions from DLS of the (a) GMO:PEO-*b*-PLA 9:1, (b) GMO:PEO-*b*-PLA 4:1, (c) GMO:PEO-*b*-PMEC 9:1, (d) GMO:PEO-*b*-PMEC 4:1 nanosystems, Figure S6. Size distributions from DLS of the (a) GMO:PEO-*b*-PLA 9:1 + resveratrol, (b) GMO:PEO-*b*-PLA 4:1 + resveratrol, (c) GMO:PEO-*b*-PMEC 9:1 + resveratrol, (d) GMO:PEO-*b*-MEC 4:1 + resveratrol nanosystems, Figure S7. Fast Fourier Transform of particles with ordered internal structure (*Pn3m* symmetry) of GMO:PEO-*b*-PLA 4:1.
