# Supplementary material for: The Influence of Hydrophobic Blocks of PEO-Containing Copolymers on Glyceryl Monooleate Lyotropic Liquid Crystalline Nanoparticles for Drug Delivery
Source: Polymers (Basel). 2021 Aug 5;13(16):2607. doi: 10.3390/polym13162607 (PMC8399356; doi:10.3390/polym13162607)
Supplement: Supplementary file 1 [file polymers-13-02607-s001.zip › polymers-1303642-supplementary.pdf]

Supporting Information

The Influence of Hydrophobic Blocks of PEO-Containing Copolymers on Glyceryl Monooleate Lyotropic Liquid Crystalline Nanoparticles for Drug Delivery

Aleksander Forys[1], Maria Chountoulesi[2], Barbara Mendrek[1], Tomasz Konieczny[1], Theodore Sentoukas[1], Marcin Godzierz[1], Aleksandra Kordyka[1], Costas Demetzos[2], Stergios Pispas[3], Barbara Trzebicka[1,*]

1. Centre of Polymer and Carbon Materials, Polish Academy of Sciences, 34 ul. M. Curie-Skłodowskiej, Zabrze, Poland; aforys@cmpw-pan.edu.pl (A.F.), bmendrek@cmpw-pan.edu.pl (B.M.), tkonieczny@cmpw-pan.edu.pl (T.K.), tsentoukas@cmpw-pan.edu.pl (T.S.), mgodzierz@cmpw-pan.edu.pl (M.G.), akordyka@cmpw-pan.edu.pl (A.K.)
2. Section of Pharmaceutical Technology, Department of Pharmacy, School of Health Sciences, National and Kapodistrian University of Athens, Panepistimioupolis Zografou, 15771, Athens, Greece; mchountoules@pharm.uoa.gr (M.C.), demetzos@pharm.uoa.gr (C.D.)
3. Theoretical and Physical Chemistry Institute, National Hellenic Research Foundation, 48 Vassileos Constantinou Avenue, 11635, Athens, Greece; pispas@eie.gr (S.P.)

Correspondence: btrzebicka@cmpw-pan.edu.pl

Polymerization procedures and chemical characterization of the block copolymers

Polymerization of poly(ethylene oxide)-*b*-poly(lactide) (PEO-*b*-PLA) was performed in an anhydrous atmosphere (glove-box, H_2O < 1 ppm, O_2 < 3 ppm). The diblock copolymer was synthesized by ring-opening polymerization (ROP) of lactide using $mPEO_{123}$-OH as an initiator and DBU as catalyst in dry CH_2Cl_2 at the monomer concentration equal to 1.000 mmol/mL at room temperature. Vial with a stirring bar was charged with a solution of LA (270.0 mg, 1 873 mmol, 10.4 eq.) in 0.937 mL of dry CH_2Cl_2. In another vial a solution of initiator $mPEG_{123}$-OH (972.0 mg, 0.180 mmol, 1 eq.) and DBU (13.7 mg, 0.09 mmol, 0.5 eq) in 0.937 mL of dry CH_2Cl_2 was prepared in next vial. Then, the initiator–catalyst solution was quickly added to the monomer solution and reaction was carried out at ambient temperature under vigorous stirring. The polymerization reaction was quenched after 40 minutes by acidification with Dowex 50WX8. The obtained copolymer was precipitated into 20-fold cold diethyl ether twice and dried under vacuum to constant weight. ^{1}H NMR spectrum of PEO-*b*-PLA is shown in figure S1a and its chromatogram in figure S2a.

In case of poly(ethylene oxide)-*b*-poly(5-methyl-5-ethyloxycarbonyl-1,3-dioxan-2-one) (PEO-*b*-PMEC), polymerization was performed in an anhydrous atmosphere (glove-box, H_2O < 1 ppm, O_2 < 3 ppm). The diblock copolymer was synthesized by ring-opening polymerization (ROP) of MEC using $mPEO_{123}$-OH as an initiator and DBU as catalyst in dry CH_2Cl_2 at the monomer concentration equal to 1.000 mmol/mL at room temperature. A vial with a stirring bar was charged with a solution of MEC (300.0 mg, 1.594 mmol, 10.0 eq.) in 0.650 mL of dry CH_2Cl_2. In another vial a solution of initiator $mPEG_{123}$-OH (860.9 mg, 0.159 mmol, 1 eq.) and DBU (12.1 mg, 0.080 mmol, 0.5 eq) in 0.950 mL of dry CH_2Cl_2 was prepared in next vial. Then, the initiator–catalyst solution was quickly added to the monomer solution and the reaction was carried out at ambient temperature under vigorous stirring. The polymerization reaction was quenched after 45 minutes by acidification with Dowex 50WX8. The obtained copolymer was precipitated into 20-fold cold diethyl ether twice and dried under vacuum to constant weight. ^{1}H NMR spectrum of PEO-*b*-PMEC is shown in figure S1b and its chromatogram in figure S2b.

NMR

^1H NMR (600 MHz) spectra were recorded using a Bruker-Avance II 600 MHz (Fremont, CA,USA) with Ultrashield Plus Magnets at r.t. in $CDCl_3$ with tetramethylsilane (TMS) as aninternal standard.

PEO-*b*-PLA, (600 MHz, $CDCl_3$, δ): 1.42 ppm (s, 3mH, CH_3), 3.38 ppm (s, 3H, CH_3-O), 3.64 ppm (s, 4nH CH_2-CH2-O), 5.16 ppm (q, mH, CH-CH_3).

PEO-*b*-PMEC, (600 MHz, $CDCl_3$, δ): 1.25 ppm (s/t, 6mH, CH_3), 3.38 ppm (s, 3H, CH_3-O), 3.64 ppm (s, 4nH CH_2-CH_2-O), 4.19 ppm (q, mH, -O-CH_2-CH_3), 4.28 ppm (m, 4mH, CH_2-C(CH_3)-CH_2).

GPC

The molar masses and molar masses dispersities of the copolymers were appointed by gel permeation chromatography.

The following column set was used: guard + GRAM 100 Å + GRAM 1000 Å + GRAM 3000 Å (Polymer Standards Service, PSS). Measurements were performed in DMF containing 5 mmol/L lithium bromide at 45 °C with a nominal flow rate of 1 mL/min. In the system a differential refractive index detector (SEC-3010 WGE Dr. Bures) was used.

The molar masses (M_n, M_w) and dispersity (M_w/M_n) were calculated based on the calibration made with poly(ethylene oxide) (PEO) standards (Polymer Standards Service, PSS).

The calibration curve was obtained on the basis of measurements of 6 commercially available PEO standards with the following molar masses: 1010, 2000, 3930, 6430, 12 140, 29 600 g/mol.

The results were evaluated using ASTRA 7 software (Wyatt Technologies).

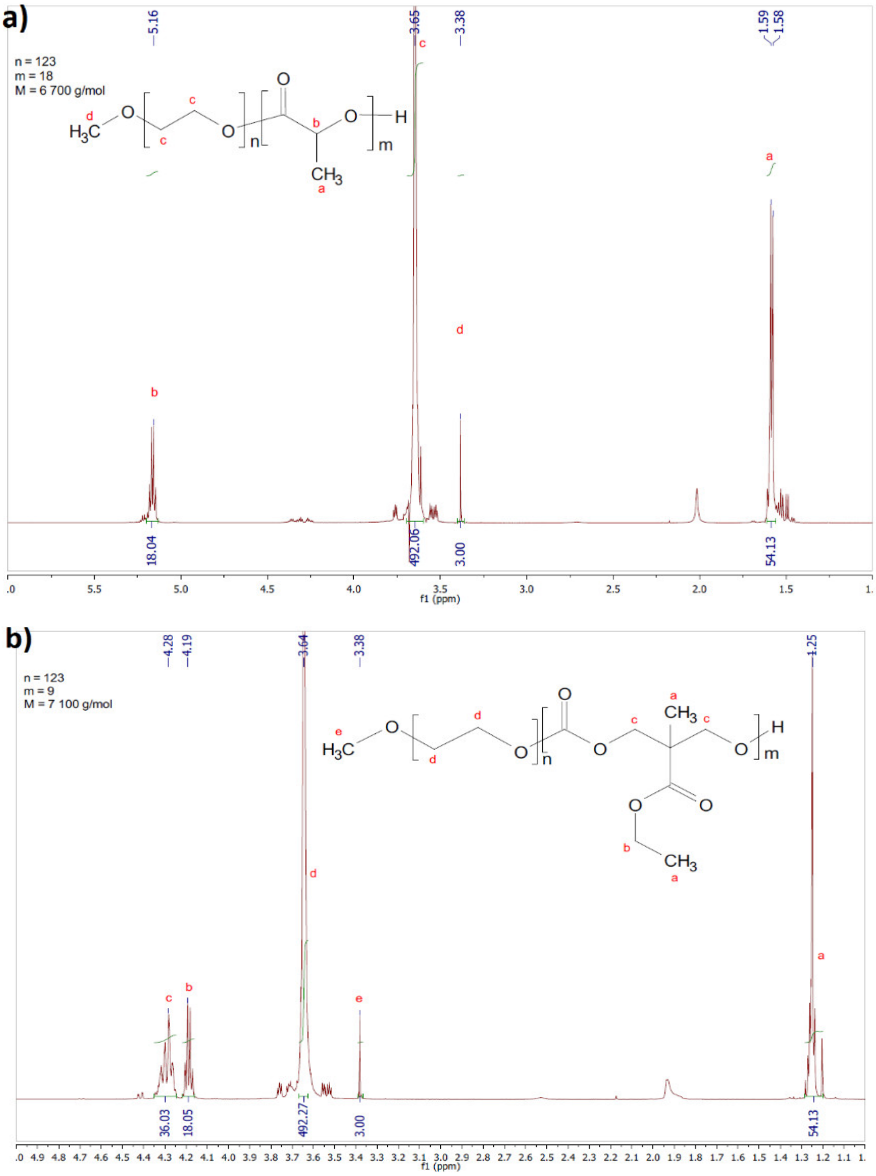

Figure S1. ^{1}H NMR spectra of the a) PEO-*b*-PLA, b) PEO-*b*-PMEC

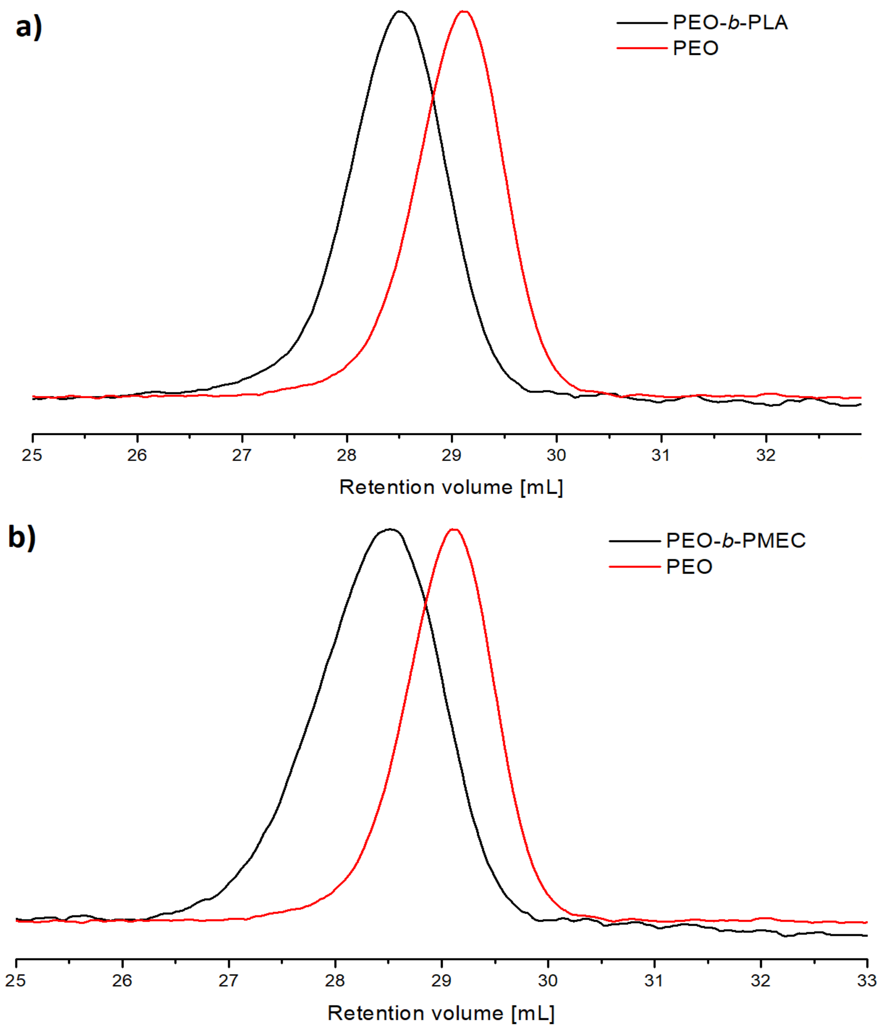

Figure S2. GPC chromatogram of the a) PEO-*b*-PLA, b) PEO-*b*-PMEC

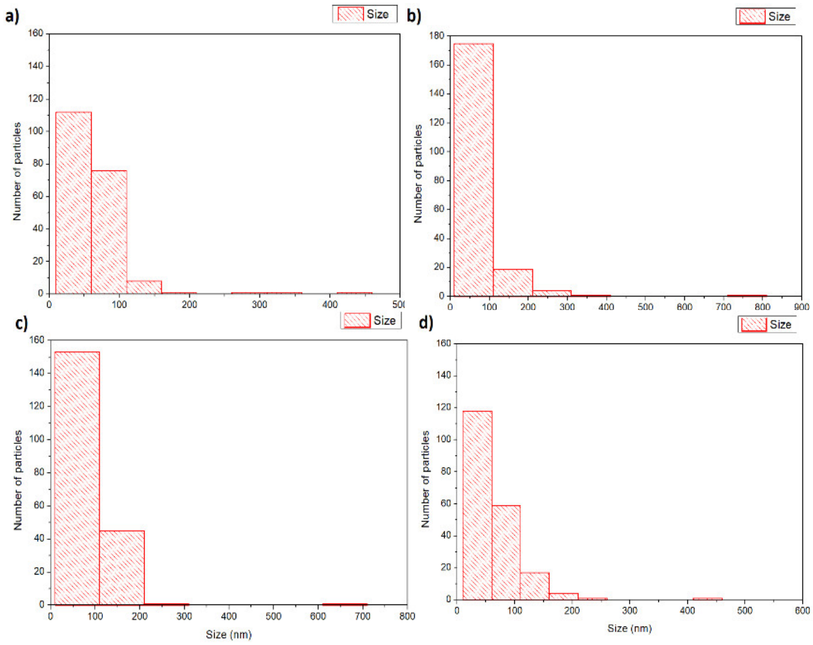

Figure S3. Cryo-TEM histograms of the a) GMO:PEO-*b*-PLA 9:1, b) GMO:PEO-*b*-PLA 4:1, c) GMO:PEO-*b*-PMEC 9:1, d) GMO:PEO-*b*-PMEC 4:1 nanosystem

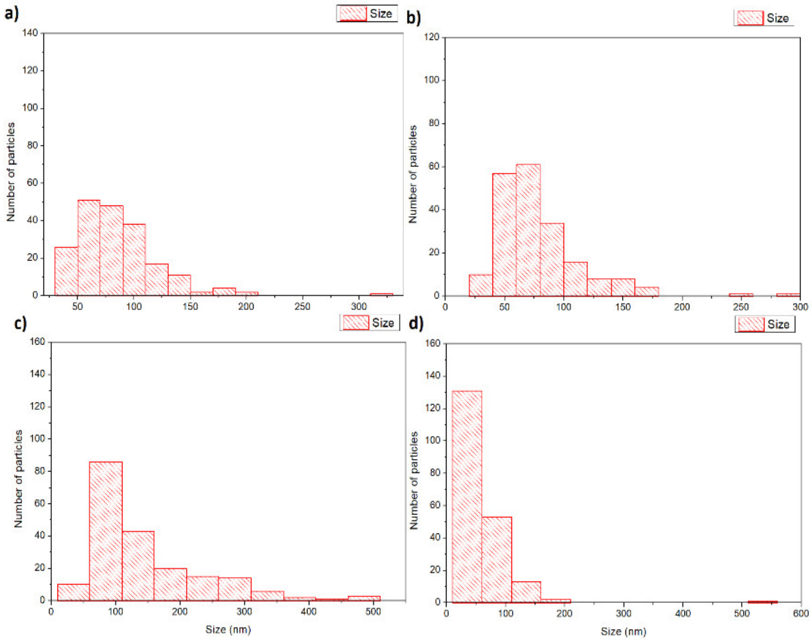

Figure S4. Cryo-TEM histograms of the of the a) GMO:PEO-*b*-PLA 9:1 + resveratrol, b) GMO:PEO-*b*-PLA 4:1 + resveratrol, c) GMO:PEO-*b*-MEC 9:1 + resveratrol, d) GMO:PEO-*b*-MEC 4:1 + resveratrol nanosystems.

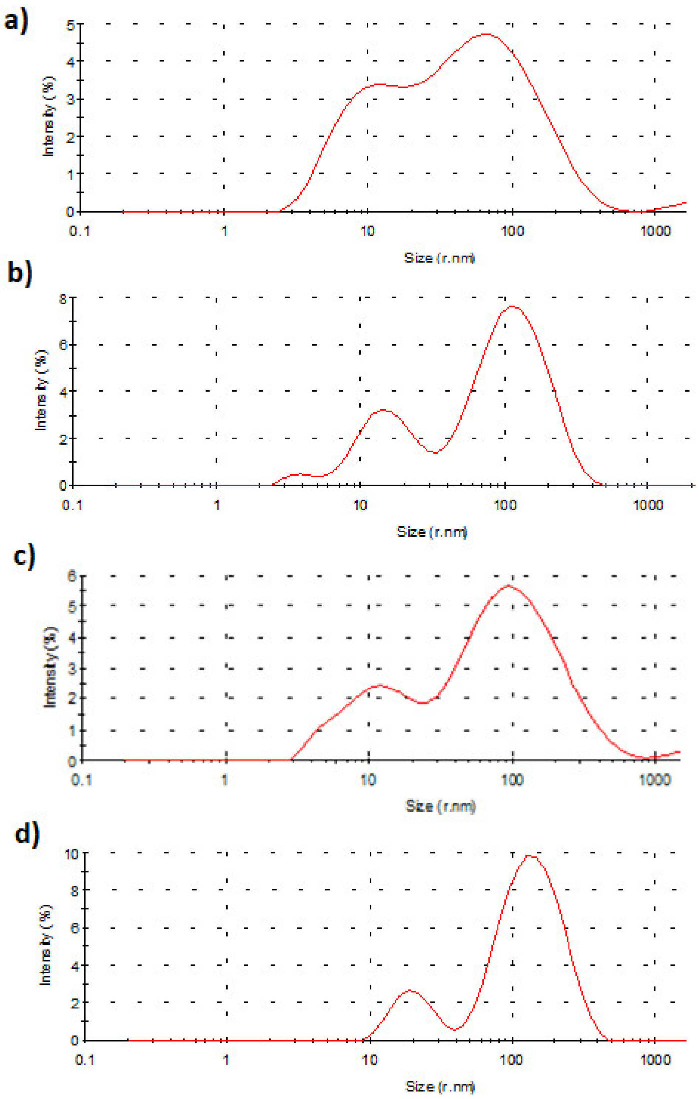

Figure S5. Size distributions from DLS of the a) GMO:PEO-*b*-PLA 9:1, b) GMO:PEO-*b*-PLA 4:1, c) GMO:PEO-*b*-PMEC 9:1, d) GMO:PEO-*b*-PMEC 4:1 nanosystems

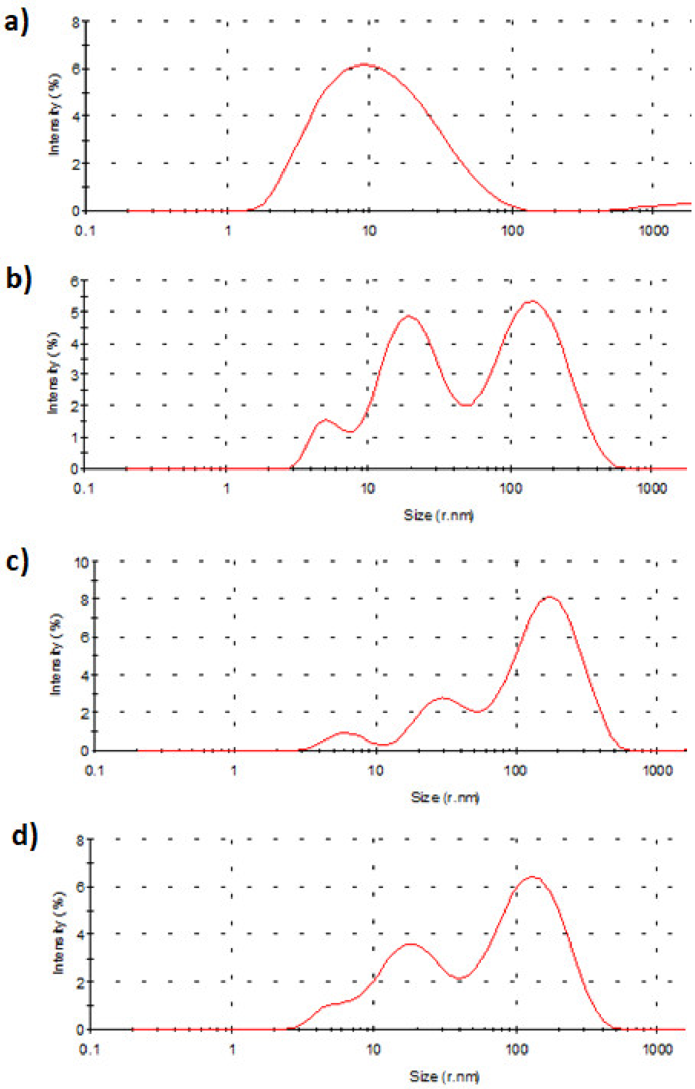

Figure S6. Size distributions from DLS of the a) GMO:PEO-*b*-PLA 9:1 + resveratrol, b) GMO:PEO-*b*-PLA 4:1 + resveratrol, c) GMO:PEO-*b*-PMEC 9:1 + resveratrol, d) GMO:PEO-*b*-MEC 4:1 + resveratrol nanosystems

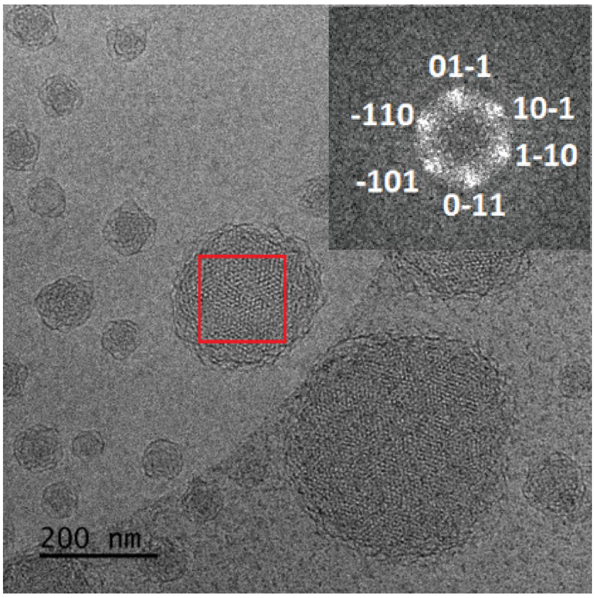

Figure S7. Fast Fourier Transform of particles with ordered internal structure (*Pn3m* symmetry) of GMO:PEO-*b*-PLA 4:1
